# Annotations

**Published:** 1892-09-17

**Authors:** 


### Annotations.

A GOOD many nurses, and a few who are not nurses, will be curious to know a little more about Nurse Emma Durham than the bare announcement in the newspapers that she has given £200 to the National Pension Fund's Benevolent Department. Nurse Durham is "only a nurse," though she is Matron at Scio House Hospital, Shanklin. That is to say, she is not a woman of wealth engaged in nursing, to whom £200 is a matter of small importance. On the contrary, the £200 represents her hard savings for several years. All the more is it to her honour that she should have had the heart to purpose, and the will to carry out her good deed. The friends of the Pension Fund, to whom Miss Durham's offer was first made, endeavoured to persuade her not to carry out what they considered an act of Quixotic generosity. It was represented to her that she had nothing to depend upon but her own earnings, and that if she must dispose of the money she had better do so by increasing her own pension. But Nurse Durham was not to be turned from her purpose. She had a pathetic kind of feeling that the money which had been earned by her in the service of the venerable laureate was not common money; it was, so to speak, Lord Tennyson's money, and it ought to be devoted to something with which his venerated name might, however indirectly, be associated. Nurse Durham valued the honour which was conferred upon her when she was selected to nurse the aged and then feeble laureate. She entered upon her duties with a spirit and resolution worthy of the highest calling. During her attendance upon the poet he completely recovered health and tone. We feel that there is a peculiar fitness in the object to which the £200 thus earned is now devoted. Nurse Durham's generosity will be long remembered, and her example may provoke many to similar deeds of loyalty and goodwill.

**A Generous Nurse.**

THE Hon. Everard Fielding has been to Lourdes, and has taken part in assisting sick persons in and out of the healing pools. Mr. Fielding went to Lourdes a moderate sceptic; he returns to this country a moderate believer in the miracles said to have been there wrought. But Mr. Fielding was not himself privileged to see one of the miracles actually wrought under his own eye. The stories told to him by persons at Lourdes were such as to make it very difficult for him to refuse credence. Mr. Fielding will find many sympathetic readers and hearers for his stories. For our part, we are glad that he should. But we cannot for all that feel actually convinced that miracles are wrought at Lourdes to-day. On the contrary, we believe they are not wrought. The miracle question is really important. There appears to be a kind of natural antagonism between scientific people and people who believe in miracles. But there is not, and cannot be, any real antagonism between miracles and science in the abstract. Science takes note of what is, and from what is it deduces what is likely to be—not, be it observed, what actually will be, but what is likely to be. Scientific men very seldom stand forth and proclaim that a thing is impossible. When they do that, they for the most part go beyond their functions and become unscientific. On the subject of modern miracles scientific men ask that there shall be rational evidence of their reality. In doing this they are performing a really important public service. An intelligent man who is truly religious loves truth above all things—truth and charity—reasonableness. A religion of this kind helps forward science amazingly. Its temper is the very temper of the noblest and most progressive science. It was Jesus Christ who said, "If thine eye be single thy whole body shall be full of light." What other motto so suitable has the world ever placed over the porch of any of its temples of science? We cannot but feel that the members of the Romish Church who encourage all the crude sensationalism and excitement which characterise the proceedings at Lourdes are to a certain extent dragging the Christian faith itself into a scoffer's arena. The honest mind insists upon truth and reality everywhere; but if there be one region more than another in which the most absolutely truthful simplicity should reign, that region is religion.

**The Lourdes Miracles?**

AT the present time the annual report of the Local Government Board for Ireland, just issued, is a document of unusual interest and importance. With the substitution of Mr. John Morley for Mr. A. J. Balfour, the temptation to inaugurate a change of methods cannot but be very strong. It is not to be believed that the various medical officers of health and parish doctors who furnish the detailed facts which are summarised in the Irish Report are influenced to any considerable extent by their political predilections; and even if they are, the presumption is that their numbers will be pretty evenly divided between the two parties. Be that as it may, a study of the actual report itself leaves the conviction upon the mind that it is like other similar reports, a cold and colourless document, dealing with statistics and facts exactly as they are. It will be generally admitted that the amount of relief given to the poor of a country in any particular year is a measure of the standard of prosperity reached by that country during that year. The compilers of the report before us (for 1891) furnish two diagrams, one of which shows by the graphic method the number of persons receiving outdoor relief each year during the past seven years, and the other, the number receiving indoor relief each year during the same period. So far as outdoor relief is concerned, the year 1885 shows a higher level of prosperity in Ireland than has ever been attained since. But the following year, 1886, was extraordinarily unprosperous. Since that time there has been a more or less steady tale of progress to tell. With some fluctuations, outdoor pauperism has continuously diminished since 1887, and the last six months of 1891-92 showed a condition of prosperity almost equal to that of the happy year 1885. But when we come to the diagram which represents the statistics of indoor pauperism, we find graphic lines of continuously increasing prosperity for the whole seven years, even including 1885. Year by year indoor pauperism steadily diminished with hardly any fluctuations at all. This is best shown by the figures of 1885 and those of 1891 respectively. In 1885 the year commenced with something over 50,000 paupers in the various workhouses, and ended with 50,000. 1891 commenced with something over 43,000, and ended with a little over 44,000. These facts speak for themselves. The potato famine of last year seems to have been tided over with but little permanent injury to the country. Nearly £300,000 were advanced to the occupiers of land for the purchase of seed potatoes. Relief works on a more or less extensive scale were started in sixty or more of the impoverished sanitary districts, and at a total cost of something under £200,000, were effectual for their purpose. As showing that this prosperity was real and not apparent the report points out that there was a large decrease in the number of persons requiring gratuitous medical treatment at their own homes and at the public dispensaries throughout the country. The total decrease in 1891 for the provinces of Ulster, Munster, Leinster, and Connaught amounted to close upon 43,000 persons. Space does not permit us to go further into the report at present, but as a statistical and impartial setting forth of actual facts, not accumulated for political purposes, its value at this critical moment cannot but be very great.

